# Open-Cage Copper Complexes Modulate Coordination and Charge Transfer

**DOI:** 10.1021/acs.inorgchem.4c01046

**Published:** 2024-06-19

**Authors:** Eric Firestone, Richard Staples, Thomas W. Hamann

**Affiliations:** Department of Chemistry, Michigan State University, East Lansing, Michigan 48824-1322, United States

## Abstract

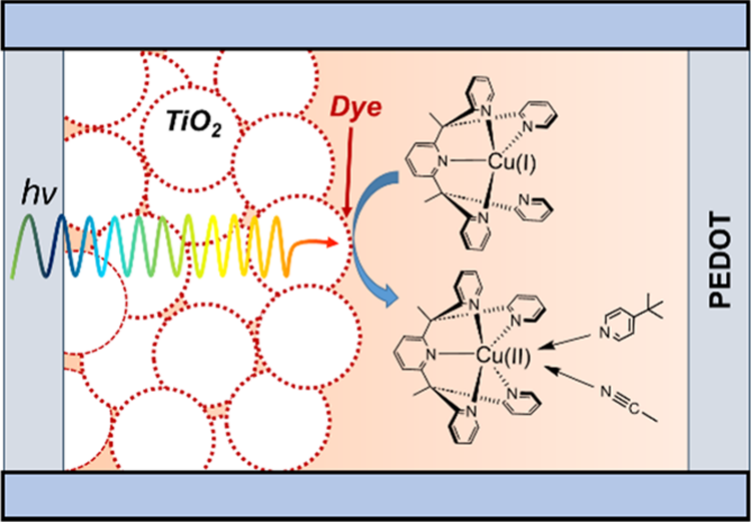

This study presents a novel copper-based redox shuttle that employs the PY5 pentadentate polypyridyl ligand in a dye-sensitized solar cell (DSSC). The [Cu(PY5)]^2+^ complex exhibits a unique five-coordinate square pyramidal geometry, characterized by a strategically labile axial position, to facilitate efficient dye regeneration while minimizing electron recombination, thereby enhancing DSSC performance. Notably, the inclusion of 4-*tert*-butylpyridine (TBP) as an additive is shown to significantly modulate the electrochemical and photophysical properties of the copper complexes, attributed to its coordination to the vacant axial site. This interaction leads to an improved open-circuit voltage and overall device efficiency, with the complexes achieving promising efficiencies under standard solar irradiance. The findings underscore the potential of utilizing copper-based redox shuttles with designed ligand geometries to overcome the limitations of current DSSC materials, opening new avenues for the design and optimization of solar energy conversion devices. This work not only contributes to the fundamental understanding of the behavior of copper complexes in DSSCs but also paves the way for future research aimed at exploiting the full potential of such geometrical and electronic configurations for the development of more robust and efficient solar energy solutions.

## Abstract 2

A five-coordinate Cu(II) complex was shown to enable weak (labile) coordination by exogenous Lewis bases to the open axial site. As a result, these copper complexes act as stable redox shuttles in dye-sensitized solar cells that allow subtle tuning of properties by the identity of the coordinating base, solvent, or counterion.

## Introduction

Following Grätzel’s seminal 1993 report on a 10% dye-sensitized solar cell (DSSC),^1^ further significant advances in efficiency were hindered by the reliance on the I_3_^–^/I^–^redox shuttle.^2−5^ The large overpotential required for efficient dye regeneration was a major limitation. Recent progress with outer-sphere redox shuttles has offered a solution to reduce the overpotential penalty and improve the DSSC performance. For example, copper-based redox shuttles have enabled 15.2% efficiency under standard solar irradiance and an astonishing 34.5% efficiency under indoor fluorescent lighting at 1000 lx intensity to be achieved.^6,7^

These high efficiencies with copper-based redox shuttles can be attributed to their combination of fast dye regeneration kinetics, indicative of low electron transfer reorganization energy, and slow recombination via back electron transfer, allowing quantitative charge collection and high open-circuit photovoltages, *V*_OC_.^6,8−15^One reason for the slow recombination is coordination of exogenous Lewis base additives to the electrolyte, including 4-*tert*-butylpyridine (TBP), to the Cu(II) species.^16,17^This can be understood since Cu(II), d^9^, complexes prefer six-coordinate (octahedral or tetragonal), five-coordinate (square pyramidal or trigonal bipyramidal), or four-coordinate (square planar) geometries, while Cu(I), *d*^10^, complexes prefer four-coordinate tetrahedral geometries.^18−21^TBP has been shown to coordinate to an open position of the Cu(II) species and sometimes substitute polydentate ligands completely.^16,17^ Lewis bases have long been employed as electrolyte additives in DSSCs to increase the performance of the devices by shifting in the titania conduction band edge to a more negative potential and blocking recombination by adsorbing to the titania surface,^22,23^ but have a more significant impact on electrolyte and overall device performance with copper redox shuttles.

Recent developments in copper-based redox shuttle design by Sun and colleagues include use of pentadentate ligands to inhibit coordination and ligand substitution by the exogeneous bases.^24^ For example, the pentadentate Cu(II) complex, [Cu(tpe)]^2+/+^ (tpe = *N*-benzyl-*N*,*N*′,*N*′-tris(pyridin-2-ylmethyl)ethylenediamine), was shown to have resistance to substitution, even when exposed to TBP. This resistance to ligand substitution is attributed to two factors. First, the increased denticity of the ligand translates to a large stability constant of the metal complex owing to the chelating effect. Second, they specifically designed the coordination sphere’s steric constraints to shield the copper complexes—particularly their oxidized forms, from TBP coordination.

In a similar vein, we recently reported a Cu complex featuring a hexadentate ligand, bpyPY4 (6,6′-bis(1,1-di(pyridine-2-yl)ethyl)-2,2′-bipyridine), to improve the stability of Cu(II) complex via the chelate effect.^25^ We found that the bpyPY4 ligand provided a dynamic coordination environment, where a 5-coordinate Cu(II) complex is formed and the noncoordinated pyridyl moiety blocks TBP coordination. In this work, we build upon the family of five-coordinate Cu(II) complexes as redox shuttles by utilization of the pentadentate ligand, 2,6-bis[1,1-bis(2-pyridyl)ethyl]pyridine (PY5), with copper metal centers. A unique feature of this geometrically constrained ligand is that it should leave an open coordination site for an exogenous base but form a stable complex via the chelation effect. Synthesis, characterization, and analysis of the behavior of these interesting copper complexes in DSSC are presented below.

## Results and Discussion

**Figure 1:**
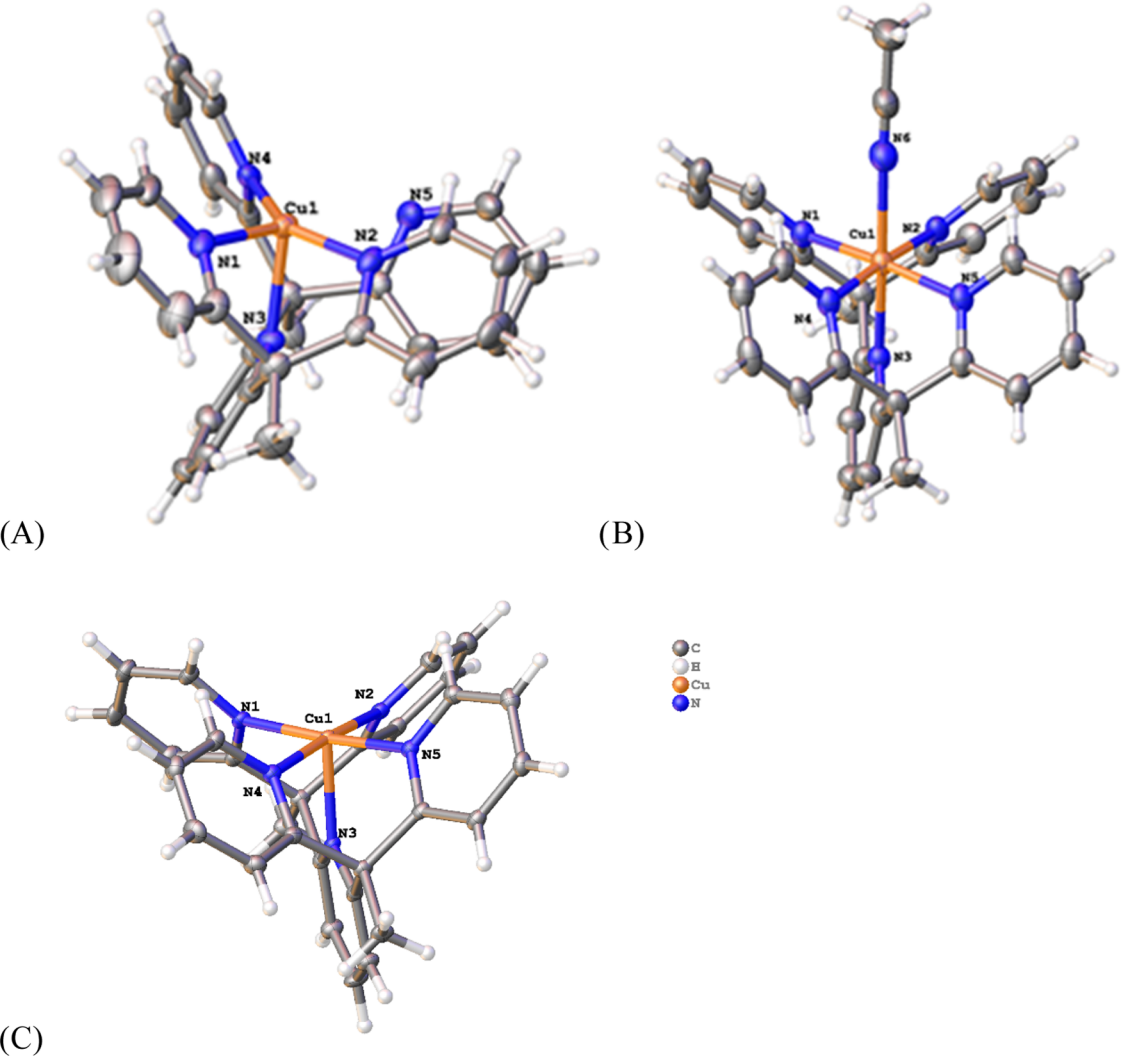
Crystal structures of the cations of (A) [Cu(PY5)]OTf, (B) [Cu(PY5)ACN]OTf_2_, and (C) [Cu(PY5)]TFSI_2_. Depicted ellipsoids are at the 50% probability level. The noninteracting anions were omitted for clarity.

The synthesis of the PY5 ligand was previously reported and the method reproduced here.^26^ The copper complexes were synthesized by reacting equimolar ratios of the PY5 ligand with copper precursors leading to the formation of [Cu(PY5)]OTf, where OTf is trifluoromethanesulfonate, and [Cu(PY5)]OTf_2_ as described in the Experimental Methodssection. The complexes were purified via recrystallization from acetonitrile (ACN) for Cu(I) complexes and dichloromethane (DCM) for Cu(II) complexes and characterized by ^1^H NMR spectroscopy and elemental analysis. Single crystals were also isolated, and X-ray diffraction revealed the solid-state structures of all complexes, as shown in Figure 1. Select bond lengths and bond angles of the structures depicted in Figure 1 are provided in Tables 1 and 2.

**Table 1:** Metal-to-Ligand Bond Distances (Å) from Single-Crystal X-ray Diffraction Data

complex	[Cu(PY5)]OTf	[Cu(PY5)ACN]OTf_2_	[Cu(PY5)]TFSI_2_.
Cu^1^–N^1^	2.100(4)	2.082(3)	2.0262(14)
Cu^1^–N^2^	1.980(4)	2.035(3)	2.0491(14)
Cu^1^–N^3^	2.057(3)	2.160(3)	2.1130(14)
Cu^1^–N^4^	1.938(4)	2.038(3)	2.0675(14)
Cu^1^–N^5^	—	2.090(3)	2.0224(14)
Cu^1^–N^6^	—	2.369(3)	—

**Table 2:** Select Bond Angles (Degrees) from Single-Crystal X-ray Diffraction Data

complex	[Cu(PY5)]OTf	[Cu(PY5)ACN]OTf_2_	[Cu(PY5)]TFSI_2_.
N^1^–Cu^1^–N^2^	90.51(15)	82.47(11)	82.32(6)
N^1^–Cu^1^–N^3^	90.20(14)	87.45(10)	91.74(6)
N^2^–Cu^1^–N^3^	88.69(15)	87.50(11)	97.08(6)
N^2^–Cu^1^–N^4^	150.50(16)	176.29(11)	175.30(6)
N^4^–Cu^1^–N^3^	97.32(14)	89.23(11)	87.35(6)
N^5^–Cu^1^–N^3^	—	87.98(11)	92.78(6)
N^3^–Cu^1^–N^6^	—	177.83(11)	—

The [Cu(PY5)]OTf complex is four-coordinate, with one of the pyridine arms in the PY5 ligand turned away from the Cu(I) center, but the steric ligands prevent formation of the preferred tetrahedral geometry. A geometric index, τ4, can be used for four-coordinate complexes to determine how distorted are from the ideal tetrahedral and square planar geometries,^27^ where a τ4 of 1 represents an ideal tetrahedral geometry and 0 represents an ideal square planar geometry. The [Cu(PY5)]OTf complex exhibited a calculated τ4 value of 0.65 which is indicative of a seesaw structure.^27^ The constrained nature of the ligand leads to a significant portion of the Cu(I) center being solvent-exposed. The choice of solvent during synthesis thus plays an important role in controlling disproportionation. When the complex was synthesized in DCM, Cu(I) disproportionated to form copper metal and a Cu(II) complex. Interestingly when an NMR was taken of the disproportionated Cu(II) product, it did not match the synthetic [Cu(PY5)]^2+^ spectrum. Therefore, we employed ACN as the solvent of choice because the Cu(I) complex did not undergo disproportionation, likely due to the stabilization of the open site by the coordinating solvent.

The solid-state structure of [Cu(PY5)]OTf_2_ shows a pseudo-octahedral geometry, with the PY5 ligand coordinated at five sites and ACN, the solvent used for synthesis, coordinated at the sixth site. The average bond length for the PY5 ligand is 2.081 Å, and the bond length between the copper and nitrogen on the ACN is 2.369 Å. In an attempt to see how the system changes when there was a vacant axial site, the crystals were regrown in the presence of a noncoordinating solvent, DCM, which revealed an interaction between one of the oxygens on the OTf counterion and the copper center in the axial position with an apparent bond length of 2.453 Å. Challenges arose in purifying ACN-bound Cu(II) complexes due to the apparent lability of ACN, leading to a mixture of copper complexes with and without ACN-bound. These observations all suggest a weakly bound, labile coordination to the open coordination site on Cu(II) complexes.

To assess the structural nuances of the copper complex, ^1^H NMR spectroscopy was employed. The ^1^H NMR spectra for the [Cu(PY5)]OTf complex, shown in Figure S1, were recorded in deuterated ACN at room temperature. The spectrum displays an integration for the 25 protons. Four peaks representing the pyridine “arms” are observed: one at approximately 8.5 ppm and three others between 7.35 and 7.95 ppm, with each integrating to four protons. Peaks at around 8.00 ppm, integrated to one proton, and 7.25 ppm, integrated to two protons, correspond to the central pyridine. The peak at approximately 2.20 ppm, integrating to six protons, is attributed to the ligand’s methyl groups. The Cu(II) complex is a paramagnetic compound making it hard to determine accurate quantitative information from the ^1^H NMR spectra shown in Figure S1. By investigating the [Cu(PY5)]OTf_2_ complex upon the addition of TBP, shown in Figure S5, it can be seen that as TBP is added to the system, there is no indication of any unbound PY5 in solution; however, there is a change in the shift of the peaks corresponding to the copper complex. This indicates the PY5 ligand is not displaced, but a reaction occurs. Furthermore, broad peaks grow into the spectra at the expected values for the TBP ligand. The broadness of the TBP peaks could be due to a rapid exchange on the NMR time scale as the TBP complexes are binding and releasing from the copper center. The interaction that is being seen is most likely due to TBP displacing the labile ACN or OTf to form [Cu(PY5)(TBP)]^2+^.

The optical spectra of the copper complexes in ACN display peaks below 450 nm, which were attributed to π–π* absorptions from pyridine units. Metal-to-ligand charge transfer bands are observed between 450 and 500 nm for [Cu(PY5)]OTf, as shown in Figure S6. Two absorption peaks were also observed for the [Cu(PY5)]OTf_2_ complex, assigned to *d–d* transitions, at 597 nm (16,750 cm^–1^), with an extinction coefficient of 79.8 M^–1^ cm^–1^, and 909 nm (11,001 cm^–1^), with an extinction coefficient of 13.2 M^–1^ cm^–1^, as shown in Figure S7. While four transitions are expected for a *d*^9^ complex with C_2*v*_ symmetry, our observation of two *d–d* transitions is consistent with a tetragonally distorted octahedral or square pyramidal geometry with two, presumably higher energy, transitions not resolved here. The assignment is consistent with the crystal structure, where a loosely bound axial ACN or OTf is observed in the solid state and likely unbound and solvated in solution. We note that Stack and co-workers reported a strikingly similar single *d–d* transition for [Cu(PY5)(Cl)]^+^ at 623 nm with an extinction coefficient of 80 M^–1^ cm^–1^.^28^ In this case, Cl occupies an equatorial position with an open axial site, with one of the pyridine arms in the PY5 ligand turned away from the Cu(II) center, to form a five-coordinate Cu(II) complex with a square pyramidal geometry. Anderson and co-worker recently reported another structurally similar Cu(II) complex with the pentadentate 2,6-(bis(bis-2-*N*-methylimidazolyl)phosphino)pyridine ligand, whose absorption spectrum is also similar to that observed here, with two apparent *d–d* transitions at approximately 600 and 900 nm (maxima and extinction coefficients not reported).^29^ The very similar *d–d* transitions observed for the structurally similar but different equatorial ligand environments, is surprising.

**Figure 2:**
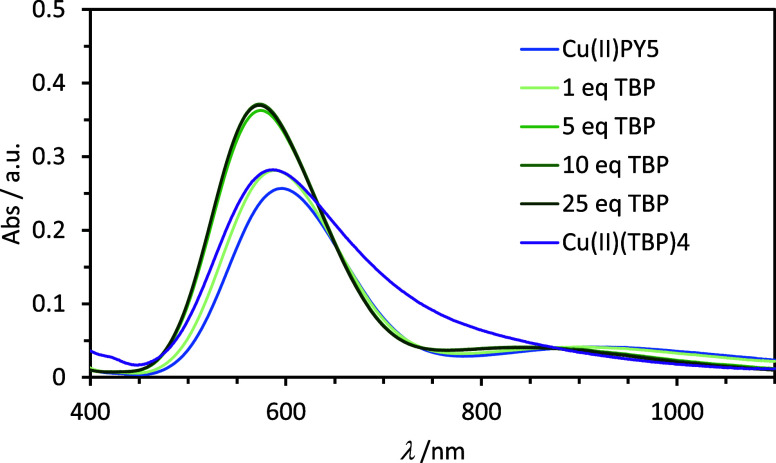
Absorbance spectra of 3.98 mM [Cu(PY5)](OTf)_2_ in anhydrous ACN with TBP titrated with increasing equivalents.

When TBP is added to the [Cu(PY5)]OTf_2_ solution, a noticeable blue shift of 23 nm (671 cm^–1^) for the peak at ∼600 nm, which also comes with an almost 50% increase in absorbance, and 58 nm (749 cm^–1^) for the peak at ∼900 nm, with minimal change in absorbance, are shown in Figure 2. This change in the spectra confirms that there is a reaction occurring between the [Cu(PY5)]OTf_2_ and TBP. Equilibrium is reached with 10 equiv of TBP relative to the Cu(II) in solution, evidenced by the absorption peaks not changing with additional aliquots of TBP. We hypothesize the reaction is TBP coordinating to the open sixth coordination site on the Cu(II) center, or displacing the weakly bound ACN/OTf. We have previously reported the displacement of bidentate ligands from Cu(II) centers by TBP to form [Cu(TBP)_4_]^2+^.^16^ The spectrum of [CuPY5]^2+^ titrated with TBP does not match the spectrum of [Cu(TBP)_4_]^2+^, shown in Figure 2 for comparison, indicating that this is not the product of titration and the PY5 ligand is still bound to the Cu(II) center, consistent with NMR spectroscopy results above. The difference spectra between the parent [Cu(PY5)]OTf_2_ complex and the titrated solutions show isosbestic points at 655, 735, and 880 nm indicating the spectra are composed of two absorbing species, as shown in Figure S8. The apparent blue shift of the spectra upon titration of TBP is consistent with the formation of a new complex assigned as [Cu(PY5)TBP]^2+^.

Cyclic voltammetry measurements were performed to determine the electrochemical potential of the parent complex and assess the effect of TPB on the redox behavior, as shown in Figures S8 and S9. In the [Cu(PY5)]OTf_2_ complex, two redox waves were observed: one at −0.372 V vs Fc^+^/Fc and another smaller wave at −0.662 V vs Fc^+^/Fc. When the complex was measured in the absence of ACN, using DCM as the solvent, the wave at −0.662 V vs Fc^+^/Fc was not observed but appeared when ACN was titrated into the solution. This indicates that the wave at −0.662 V vs Fc^+^/Fc corresponds to the ACN-bound complex, and the peak at −0.372 V vs Fc^+^/Fc is either the OTf bound complex or a 5-coordinate [Cu(PY5)]^2+^ complex. In order to test these possibilities, the copper complex was synthesized with bistrifilmide (TFSI) as the counterion, which is noncoordinating, and measured in anhydrous ACN. A single wave was observed at −0.382 V vs Fc^+^/Fc, which shows this wave cannot be due to bound OTf, and we thus assign it to the 5-coordinate [Cu(PY5)]^2+^ complex. To further test the possibility that OTf is bound to the copper center in solution, a variable-temperature ^19^F NMR spectroscopy experiment was conducted, which involved cooling the solution from room temperature to −40 °C. Only a single peak is observed, whereas two peaks are expected if one OTf is bound and one is in the outer coordination sphere. Upon lowering the temperature, the fluorine peak corresponding to the triflate counterion exhibits a decrease in intensity coupled with an increase in sharpness; see Figures S10–S11. This observation suggests that the counterion does not undergo exchange with the axial site on the copper center, and the counterion does not interact with the copper center in solution. Such an exchange would typically result in the broadening of the peak as the temperature decreases. Notably, this trend remained consistent irrespective of whether ACN or DCM was used as the solvent. When this evidence is compiled with the previous results of the ultraviolet–visible (UV–vis) and cyclic voltammetry (CV), it becomes clear that the solution geometry of the [Cu(PY5)]^2+^ complex is square pyramidal.

**Figure 3:**
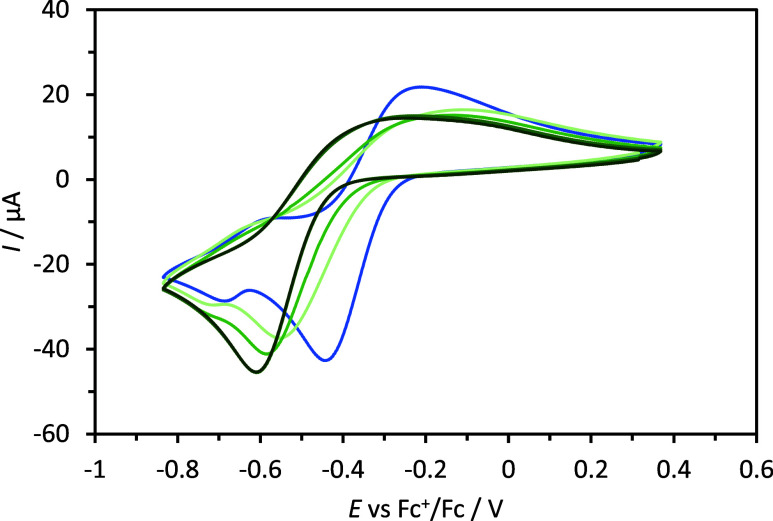
Cyclic voltammogram of 3.98 mM [Cu(PY5)]OTf_2_ in anhydrous ACN with 0.1 M TBAPF_6_ using a glassy carbon working electrode (blue). CVs with the addition of 1 equiv of TBP (pale green), 5 equiv of TBP (green), 10 equiv of TBP (deep green), and 15 equiv of TBP (dark green) are also shown.

Upon addition of TBP to the solutions containing [Cu(PY5)]OTf_2_ or [Cu(PY5)]TFSI_2_, the redox waves dissipate and a new wave grows at −0.512 V vs Fc^+^/Fc. This new wave continues to grow as TBP is added until ca. 10 equiv relative to the amount of Cu(II) in the solution is reached, where it is the only redox wave observed and is constant, as shown in Figure 3. This is the same end point that can be determined from the absorption spectra, demonstrating that they both derive from forming the same complex in solution, which has gone to completion and is assigned to coordinated TBP to the open site. Attempts to isolate the TPB complex were unsuccessful, however. The anodic wave is very broad, with a poorly defined peak. The detailed reason for this unusual waveform is still not clear but likely due to the coordination of TBP coupled with oxidation of the Cu(I) species. Thus, 10 equiv of TBP were added to the electrolyte in all cells investigated in this paper, where there should be negligible mixtures of coordination complexes, as described below.

The solution potentials were determined with open-circuit potential measurements by using a Pt wire. The solution potential is similar between the OTf and TFSI versions of the complex, −0.372 and −0.398 V vs Fc^+^/Fc, respectively, which are both slightly negative of the predicted Nernstian potentials of −0.357 and −0.365 V vs Fc^+^/Fc, respectively. This indicates that the predominant redox shuttle that is affecting the devices is the one represented by the wave at ca. −0.375 V vs Fc^+^/Fc, with minimal contribution from the wave at −0.662 V vs Fc^+^/Fc. When TBP is added to the devices, the solution potential shifts negatively by ca. 130 mV to −0.486 and −0.501 V vs Fc^+^/Fc for the OTf and TFSI complexes, respectively, which matches well with the predicted −0.503 V vs Fc^+^/Fc. While the change of counterion has some effect on the redox properties of the complex, the performance of the DSSC devices was unaffected by the counterion chosen, so the [Cu(PY5)]OTf_2_ was used for further studies.

The cross-exchange electron transfer rates between [Cu(PY5)]OTf_2_ and octamethylferrocene (Me_8_Fc) were measured via stopped-flow spectroscopy using methods previously reported and described in the Supporting Information.^25,30^ The self-exchange rate constant for Me_8_Fc^+/0^ was previously determined to be 2.0 (±0.4) × 10^7^ M^–1^ s^–1^ from NMR line broadening measurements.^30^ Thus, calculation of the self-exchange rate for the [Cu(PY5)]OT*f*_1/2_ couple could be determined from the Marcus cross-exchange formalism,^31^and was found to be 88.1 (±7.3) M^–1^ s^–1^. This relatively slow self-exchange rate constant is attributed to the large inner-sphere reorganization energy of approximately 0.74 eV due to the change in geometry and coordination number upon electron transfer. This self-exchange rate constant is about an order of magnitude faster, with an ∼0.25 eV lower inner-sphere reorganization energy, compared to other related cobalt and copper cage complexes that undergo a change in coordination number upon electron transfer which we attribute to the more strained geometry of the ligand preventing larger structural changes in the backbone.^25,30^ Thus, [Cu(PY5)]^+^ should be a better dye regenerator and result in high current densities.

**Figure 4:**
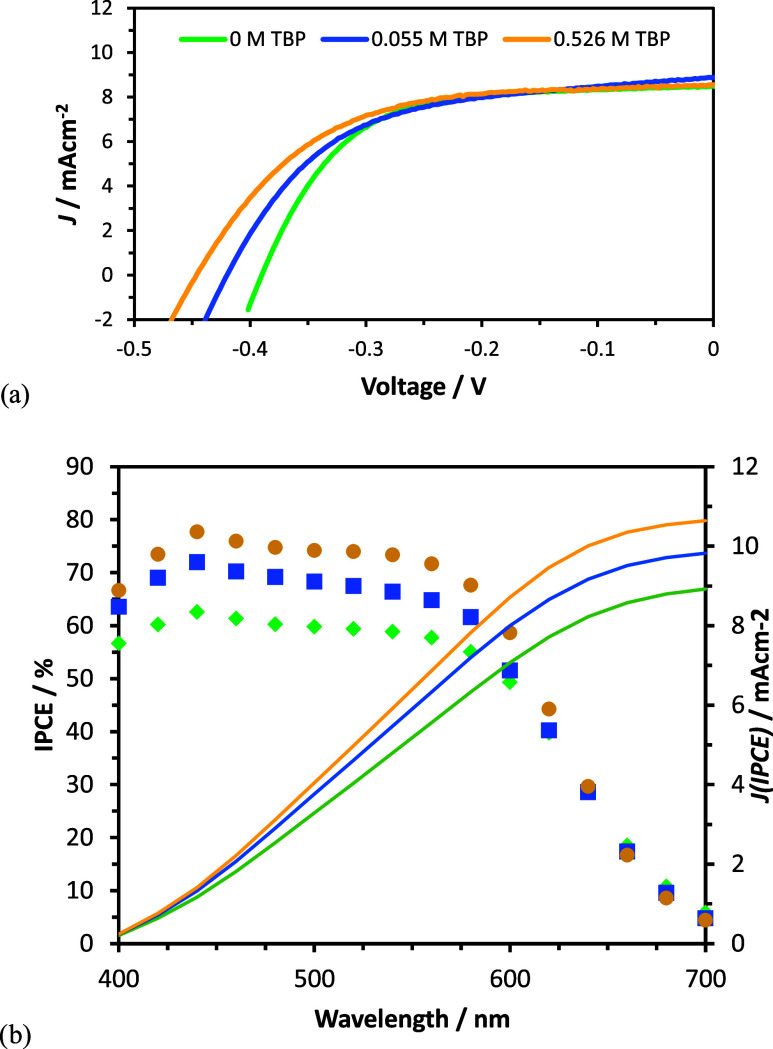
(a) Plot of *J–V* curves of DSSC devices containing 0.10 M [Cu(PY5)]OTf, 0.05 M [Cu(PY5)]OTf_2_, 0.1 M LiOTf and 0 M TBP (green), 0.055 M TBP (blue), and 0.526 M TBP (yellow) in dry ACN. (B) Plots of IPCE (symbols) and results of integrated IPCE, *J*(IPCE), to obtain predicted current density for DSSCs containing 0 M TBP (green), 0.055 M TBP (blue), and 0.526 M TBP (yellow).

**Scheme 1:**
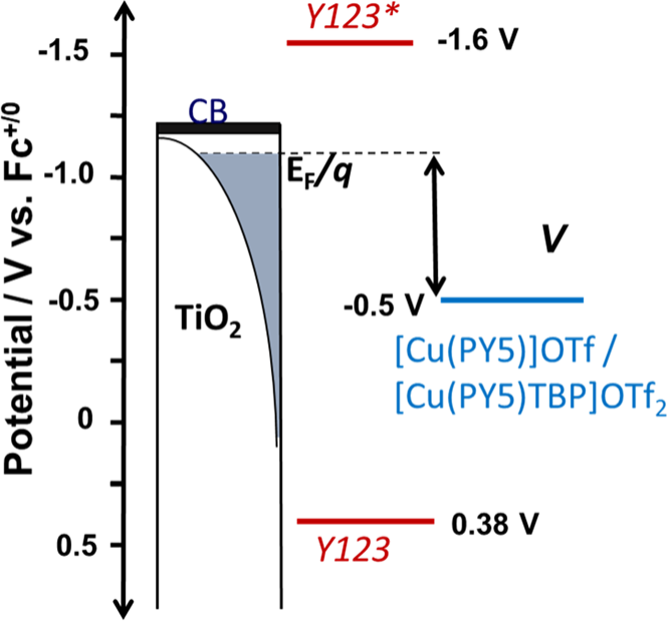
Energy Diagram of TiO_2_ Sensitized with the Y123 Dye in Contact with [Cu(PY5)]^+^/ [Cu(PY5)TBP]^2+^

The behavior of the [Cu(PY5)]^2+/+^ complexes in DSSCs and the effects of TBP were therefore investigated by fabricating devices with various concentrations of TBP. Scheme 1 depicts the energy level diagram of the DSSCs, including the TiO_2_ conduction band,^32^ Y123 dye,^33^ and the Cu(PY5) electrolyte. Figure 4a shows the current density vs applied voltage (*J–V*) curves for the best devices measured for each condition. The compiled results of all devices are provided in the Supporting Information. The performance of all devices improved with the addition of TBP; however, the effect is relatively small and primarily due to increases in open-circuit photovoltage (*V*_OC_). The steady-state short-circuit photocurrent density, *J*_SC_, is around 9 mAcm^–2^ for all devices. The incident photon-to-current efficiency (IPCE) spectra were also measured, as shown in Figure 4b. Integration of the IPCE yields a predicted *J*_SC_ under white light. The predicted current is in general agreement with the *J*_SC_ determined from *J–V* curves; however, the IPCE and integrated *J* indicate a trend of increasing photon conversion with increasing TBP concentrations, which is not observed under white light in the *J–V* results. Thus, the intrinsic kinetics giving rise to photocurrent generation improve with TPB; however, this is another process that limits the photocurrent under white light conditions. These increases in IPCE are attributed to a decreased level of recombination to the Cu(II) form of the redox shuttle as TBP is added to the solution, which improves the charge collection efficiency. This is a well-known effect of TBP which results from steric blocking of recombination from surface adsorbed TBP.^22^ TBP also increases electrolyte viscosity, which will result in mass-transport limitations of the photocurrent, which explains the essentially constant photocurrent under white light, i.e., it is limited by mass transport, not intrinsic kinetics. Interestingly, the *V*_OC_ improves by ca. 100 mV with the addition of TBP despite a ca. 130 mV negative shift in the solution potential. Since the *V*_OC_ is the difference in solution potential and Fermi level (*E*_F_) in the TiO_2_ at open circuit, this result implies that the *E*_F_ increases by ca. 230 mV with TBP. TBP is known to raise the conduction band energy and block recombination, both of which can result in a higher *E*_F_. The partitioning of these effects is challenging; however, decreased recombination with TBP is consistent with the increased IPCE which we take as the dominant effect. We note that increases in the conduction band increase the driving force, and thus the rate, of recombination, and is thus likely a minor or negligible contribution to increased *V*_OC_.

## Conclusions

Our research introduces a novel copper-based redox shuttle for dye-sensitized solar cells, utilizing a pentadentate polypyridyl ligand with a labile axial position to form [Cu(PY5)]^2+/+^ complexes with a unique five-coordinate square pyramidal geometry. This design not only stabilizes the complex but also enables precise interactions with additives like TBP, modulating the device’s electrochemical properties without displacing the PY5 ligand. These strategic interactions enhance DSSC efficiencies, may increase compatibility and optimization with new sensitizers being developed,^34^ and offer insights into optimizing redox shuttle design for improved solar energy conversion, setting a foundation for future advancements in robust and efficient solar technologies.

## Experimental Methods

### Synthesis

ACN, deuterated ACN, DCM, methanol, ethanol, diethyl ether, deionized water, 1,1-bis(2-pyridyl)ethane, 2,6-difluoropyridine, 2.5 M *n*-butyl lithium in hexanes, tetrakisacetonitrile copper(I) triflate, copper(II) triflate, silver nitrate, tetrabutylammonium hexafluorophosphate, lithium triflate, lithium bistriflimide, silver bistriflimide, copper(I) chloride, copper(I) bistriflimide, and isopropyl alcohol were purchased from Sigma-Aldrich and used as received.

The starting material, 1,1-bis(2-pyridyl)ethane, and the PY5 ligand were synthesized according to published procedures.^35^

#### [Cu(PY5)](OTf)

A mixture of PY5 (74.5 mg, 0.168 mmol) and [Cu(ACN)_4_](OTf) (57.5 mg, 0.153 mmol) in anhydrous ACN was stirred for 30 min at room temperature. The solution was precipitated with anhydrous diethyl ether, forming a yellow solid, and the solid was collected. The solid was dried under vacuum. (97.8 mg 97.4% yield) ^1^H NMR (500 MHz, ACN-d3): δ = 8.53 (d, 4H); 8.04 (t, 1H); 7.92 (d, 2H); 7.77 (t, 4H); 7.37 (d, 4H); 7.26 (t, 4H); 2.20 (s, 6H); 2.18 (1.5 H). Elem. Anal. Calc. for C_30_H_25_CuF_3_N_6_O_3_S C, 54.42; H, 3.98; N, 12.28. Found: C, 54.79; H, 3.91; N, 12.05. TOF-MS-ES+ *m*/*z* calcd for [Cu(PY5)], C_29_H_25_CuN_5_ 506.14; Found, 506.1407.

#### [Cu(PY5)](TFSI)

Copper bistrifilmide was made in situ by combining silver bistriflimide (47.0 mg, 0.121 mmol) and copper chloride (12.0 mg, 0.121 mmol) in minimal anhydrous ACN. The solution was stirred for 30 min at room temperature. After mixing, the solid silver chloride was removed via filtration, and then the copper bistrifilmide solution was added to PY5 (52.5 mg, 0.118 mmol) and stirred overnight. The solution was precipitated with anhydrous diethyl ether, forming a yellow solid, and the solid was collected. The solid was dried under vacuum (90.2 mg, 96.9% yield). ^1^H NMR (500 MHz, ACN-*d*_3_): δ = 8.53 (d, 4H); 8.04 (t, 1H); 7.92 (d, 2H); 7.77 (t, 4H); 7.37 (d, 4H); 7.26 (t, 4H); 2.20 (s, 6H); 2.18 (1.5 H). Elem. Anal. Calc. for C_31_H_25_CuF_6_N_6_O_4_S_2_ C, 54.42; H, 3.98; N, 12.28. Found: C, 54.79; H, 3.91; N, 12.05. TOF-MS-ES+ *m*/*z* calcd for [Cu(PY5)], 506.14; Found, 506.1407.

#### [Cu(PY5)](OTf)_2_

A mixture of PY5 (0.2924 g, 0.66 mmol) and Cu(OTf)_2_ (0.2210 g, 0.61 mmol) in anhydrous DCM was stirred for 30 min at room temperature. The solution was precipitated with anhydrous diethyl ether, forming a blue solid, and the solid was collected. The solid was dried under vacuum (0.4776 mg, 92.5% yield) Elem. Anal. Calc. for C_31_H_25_CuF_6_N_6_O_6_S_2_ C, 46.24; H, 3.13; N, 7.89. Found: C, 45.93; H, 3.32; N, 8.38.

#### [Cu(PY5)](TFSI)_2_

A mixture of PY5 (47.8 mg, 0.108 mmol) and Cu(TFSI)_2_ (67.2 mg, 0.108 mmol) in anhydrous DCM was stirred for 30 min at room temperature. The solution was precipitated with anhydrous diethyl ether, forming a blue solid, and the solid was collected. The solid was dried under vacuum. Any further purification was done via recrystallization from ACN with diffused ether (46.3 mg, 40.1% yield) Elem. Anal. Calc. for C_35_H_28_CuF_12_N_8_O_8_S_4_ C, 37.93; H, 2.55; N, 10.11. Found: C, 37.38; H, 2.31; N, 9.41.

### Characterization

All NMR spectra were recorded on an Agilent DirectDrive2 500 MHz spectrometer at room temperature and referenced to residual solvent signals. All NMR spectra were evaluated by using the MestReNova software package features. Cyclic voltammograms were obtained using μAutolabIII potentiostat using BASi glassy carbon electrode, a platinum mesh counter electrode, and a fabricated 0.01 M AgNO_3_, 0.1 M TBAPF_6_ in ACN Ag/AgNO_3_ reference electrode. All measurements were internally referenced to an Fc^+^/Fc couple via the addition of ferrocene to solution after measurements or run in a parallel solution of the same solvent/electrolyte. UV–vis spectra were taken using a PerkinElmer Lambda 35 UV–vis spectrometer using a 1 cm path length quartz cuvette at 480 nm/min. Elemental analysis data were obtained via Midwest Microlab. For single-crystal X-ray diffraction, single crystals were mounted on a nylon loop with paratone oil using a Bruker APEX-II CCD diffractometer. Crystals were maintained at T 1/4 173(2) K during data collection. Using Olex2, the structures were solved with the ShelXS structure solution program using the direct methods solution method. Photoelectrochemical measurements were performed with a potentiostat (Autolab PGSTAT 128N) in combination with a xenon arc lamp. An AM 1.5 solar filter was used to simulate sunlight at 100 mW cm^–2^, and the light intensity was calibrated with a certified reference cell system (Oriel Reference Solar Cell & Meter). A black mask with an open area of 0.07 cm^–2^ was applied on top of the cell active area. A monochromator (Horiba Jobin Yvon MicroHR) attached to the 450 W xenon arc light source was used for monochromatic light for IPCE measurements. The photon flux of the light incident on the samples was measured with a laser power meter (Nova II Ophir). IPCE measurements were made at 20 nm intervals between 400 and 700 nm at short-circuit current.

### Device Fabrication

TEC 15 FTO was cut into 1.5 cm by 2 cm pieces which were sonicated in soapy DI water for 15 min, followed by manual scrubbing of the FTO with Kimwipes. The FTO pieces were then sonicated in DI water for 10 min, rinsed with acetone, and sonicated in isopropanol for 10 min. The FTO pieces were dried in room air and then immersed in an aqueous 40 mM solution TiCl_4_ solution for 60 min at 70 °C. The water used for the TiCl_4_ treatment was preheated to 70 °C prior to adding 2 M TiCl_4_ to the water. The 40 mM solution was immediately poured onto the samples and placed in a 70 °C oven for the 60 min deposition. The FTO pieces were immediately rinsed with 18 MΩ water followed by isopropanol and were annealed by heating from room temperature to 500 °C, holding at 500 °C for 30 min. A 0.36 cm^2^ area was doctor-bladed with commercial 30 nm TiO_2_ nanoparticle paste (DSL 30NRD). The transparent films were left to rest for 10 min and were then placed in a 125 °C oven for 30 min. The samples were annealed in an oven that was ramped to 325 °C for 5 min, 375 °C for 5 min, 450 °C for 5 min, and 500 °C for 15 min. The 30 nm nanoparticle film thickness was 8.2 μm. After cooling to room temperature, a second TiCl_4_ treatment was performed as described above. When the anodes had cooled to 80 °C, they were soaked in a dye solution of 0.1 mM Y123 in 1:1 ACN/*tert*-butyl alcohol for 18 h. After the anodes were soaked, they were rinsed with ACN and dried gently under a stream of nitrogen.

The PEDOT counter electrodes were prepared by electropolymerization in a solution of 0.01 M EDOT and 0.1 M LiClO_4_ in 0.1 M SDS in 18 MΩ water. A constant current of 8.3 mA for 250 s was applied to a 54 cm^2^ piece of TEC 8 FTO with predrilled holes using an equal-sized piece of FTO as the counter electrode. The PEDOT electrodes were then washed with DI water and ACN before being dried under a gentle stream of nitrogen and cut into 1.5 × 1.0 cm^2^ pieces. The working and counter electrodes were sandwiched together with 25 μm Surlyn films by placing them on a 140 °C hot plate and applying pressure. The cells were then filled in a nitrogen-filled glovebox with electrolyte through one of the two predrilled holes and were sealed with 25 μm Surlyn backed by a glass coverslip and applied heat to seal with a soldering iron. The electrolyte consisted of 0.10 M Cu(I), 0.05 M Cu(II), 0.1 M Li(Counterion), and 0.5 M 4-*tert*-butylpyridine in ACN. Contact to the TiO_2_ electrode was made by soldering a thin layer of indium wire onto the FTO.

## Supporting Information Available

NMR spectra of ligand and complexes reported herein, results of electrochemical and spectroscopic measurements, self-exchange measurements, and device performance metrics (PDF)
