# Supplementary material for: Open-Cage Copper Complexes Modulate Coordination and Charge Transfer
Source: Inorg Chem. 2024 Jun 19;63(26):12081–8. doi: 10.1021/acs.inorgchem.4c01046 (PMC11220750; doi:10.1021/acs.inorgchem.4c01046)
Supplement: Supplementary file 1 — ic4c01046_si_001.pdf [file ic4c01046_si_001.pdf]

Supporting Information

Open-Cage Copper Complexes Modulate Coordination and Charge Transfer

Eric Firestone, Richard Staples and Thomas W. Hamann*

Department of Chemistry, Michigan State University, East Lansing, Michigan 48824-1322, United States

*hamann@chemistry.msu.edu

Scheme S1: Syntheses of the $[Cu(PY5)]^{2+/+}$ complexes, where X is the counter ion used for each batch, either triflate (OTf) or bistriflimide (TFSI).

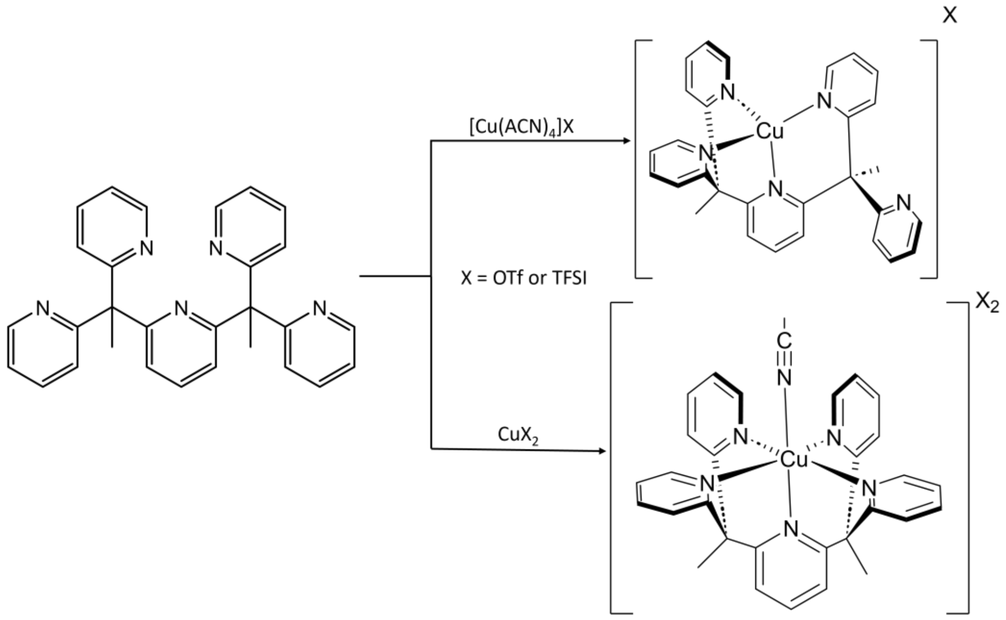

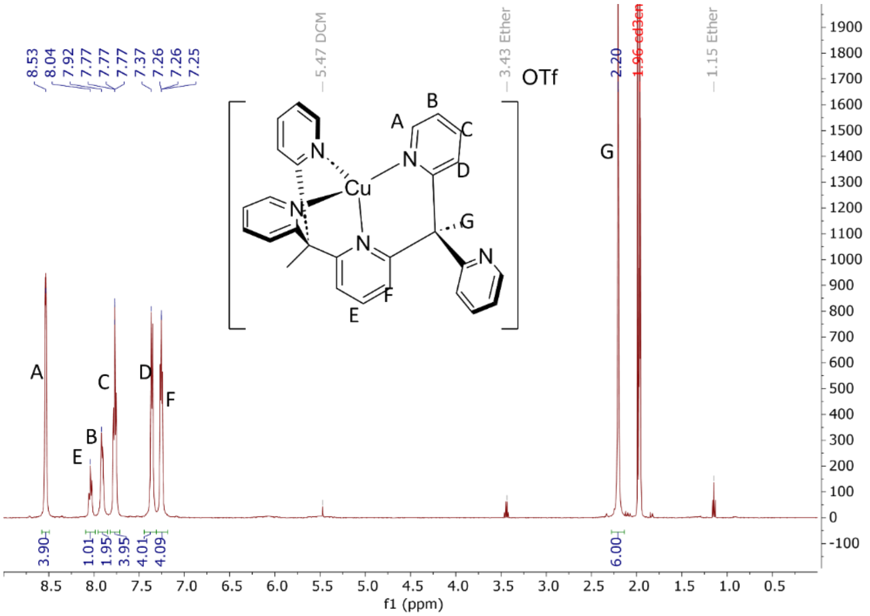

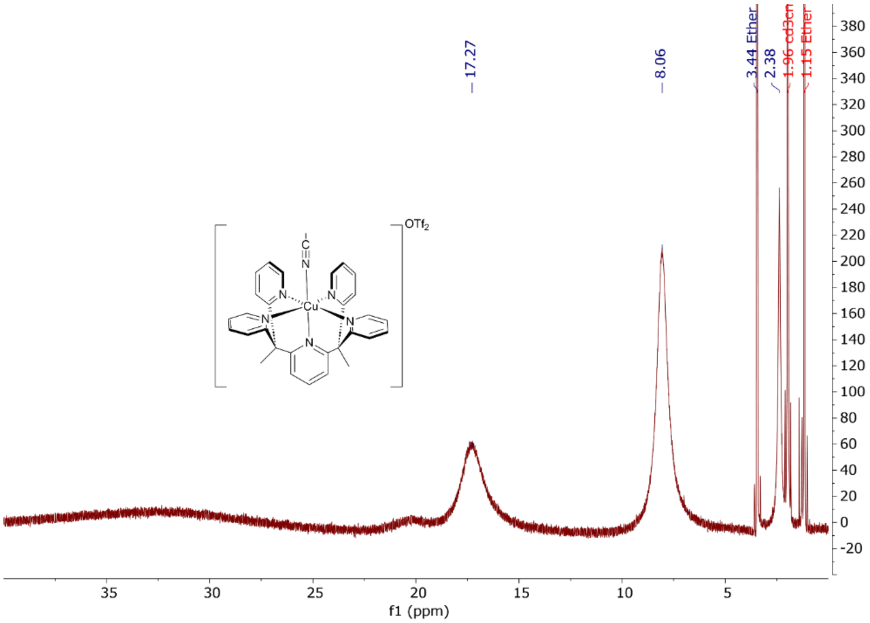

Figure S1. Top: ^{1}H NMR spectrum of [Cu(PY5)]OTf in CD_3CN. Bottom: ^{1}H NMR spectrum of [Cu(PY5)ACN]OTf_2 in CD_3CN.

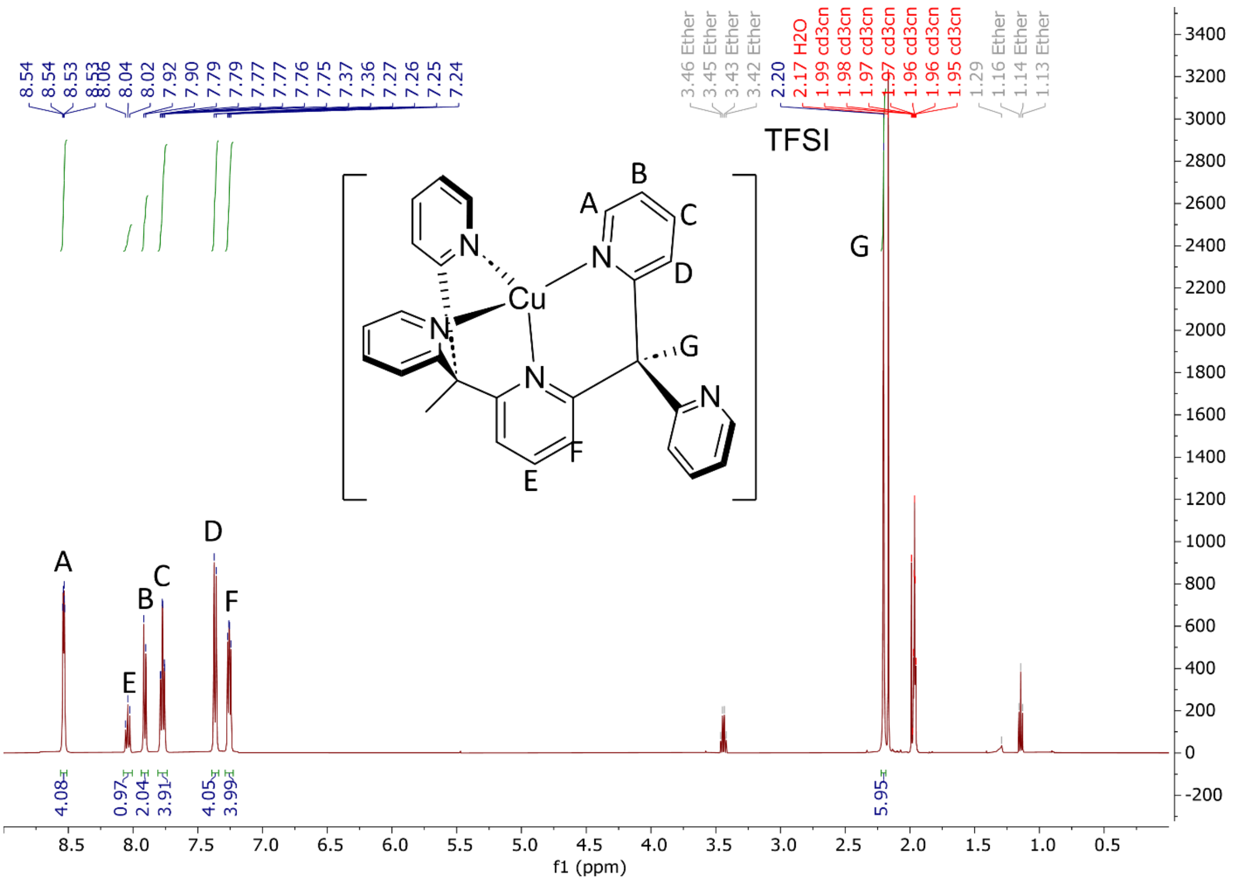

Figure S2: ^{1}H NMR spectrum of [Cu(PY5)]TFSI in CD_3CN on a 500 MHz instrument.

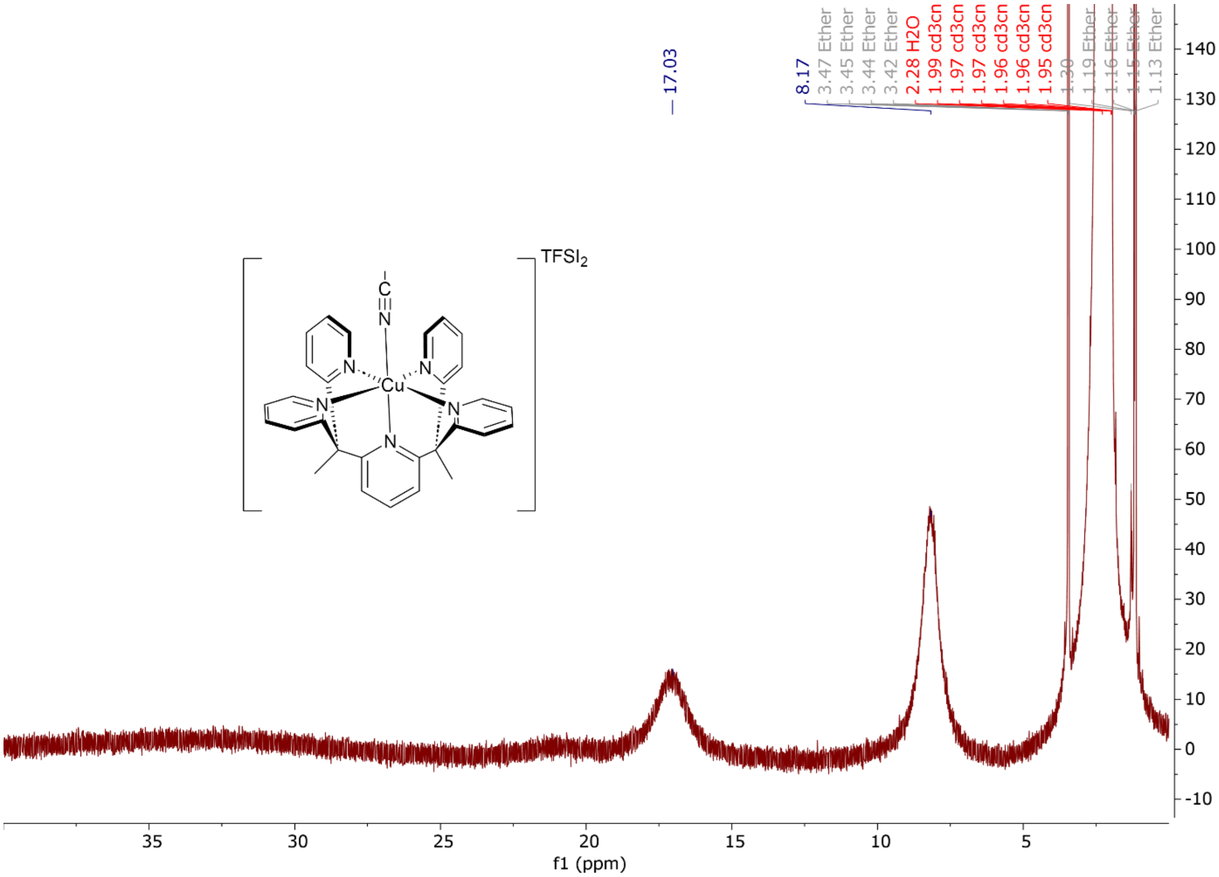

Figure S3: ^{1}H NMR spectrum of $[Cu(PY5)]TFSI_2$ in CD_3CN on a 500 MHz instrument.

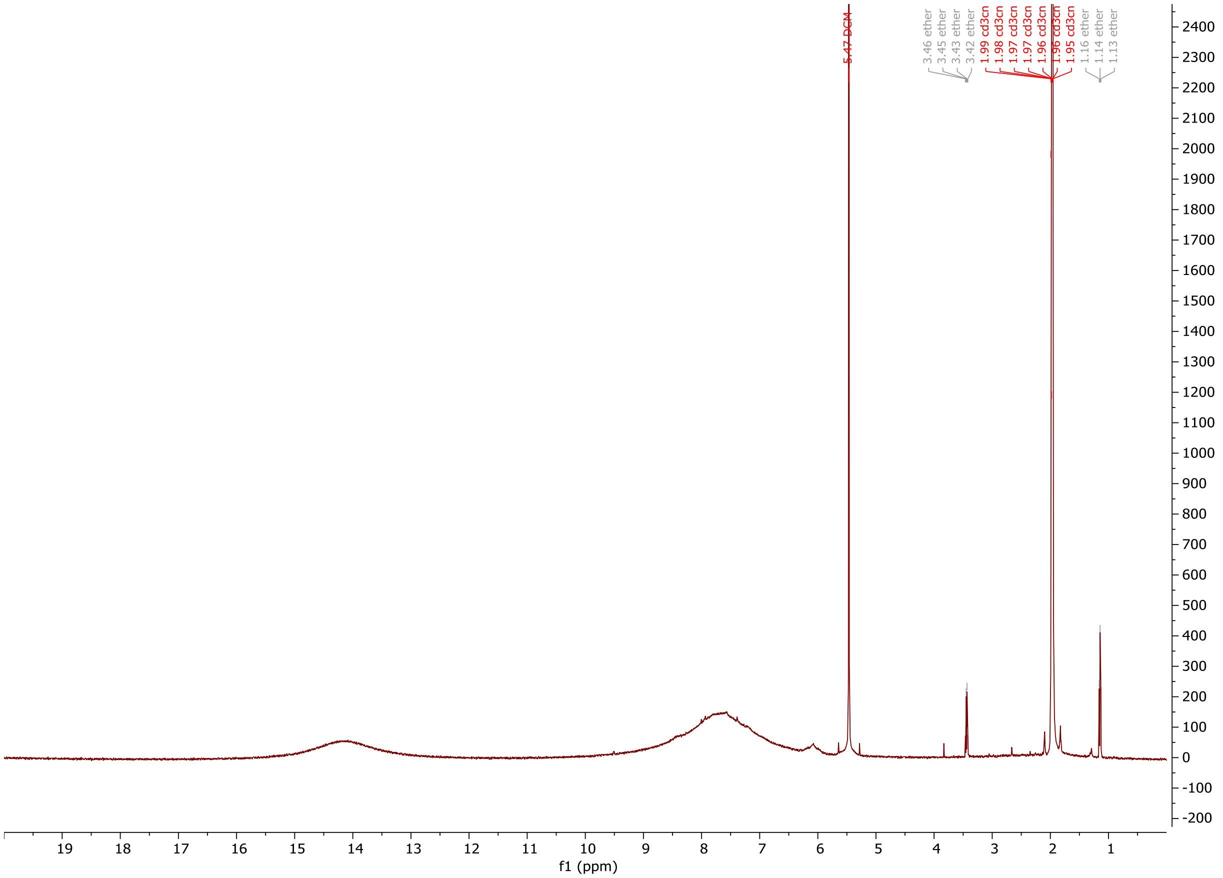

Figure S4: ^{1}H-NMR spectrum of disproportionated [Cu(PY5)]OTf_2 in deuterated acetonitrile.

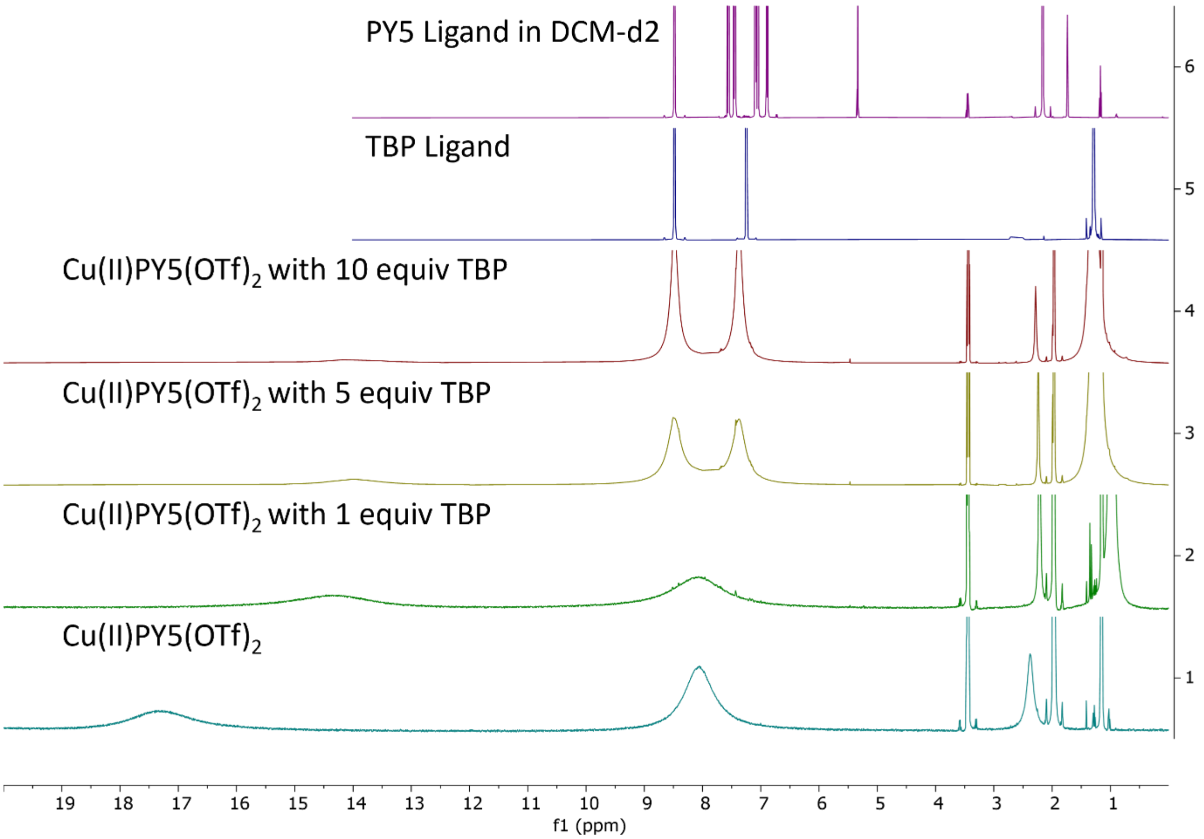

Figure S5: ^{1}H-NMR spectra of 12 mM of [Cu(PY5)]OTf_2 with increasing equivalents of TBP added to the solution, all spectra were taken using anhydrous acetonitrile-d3.

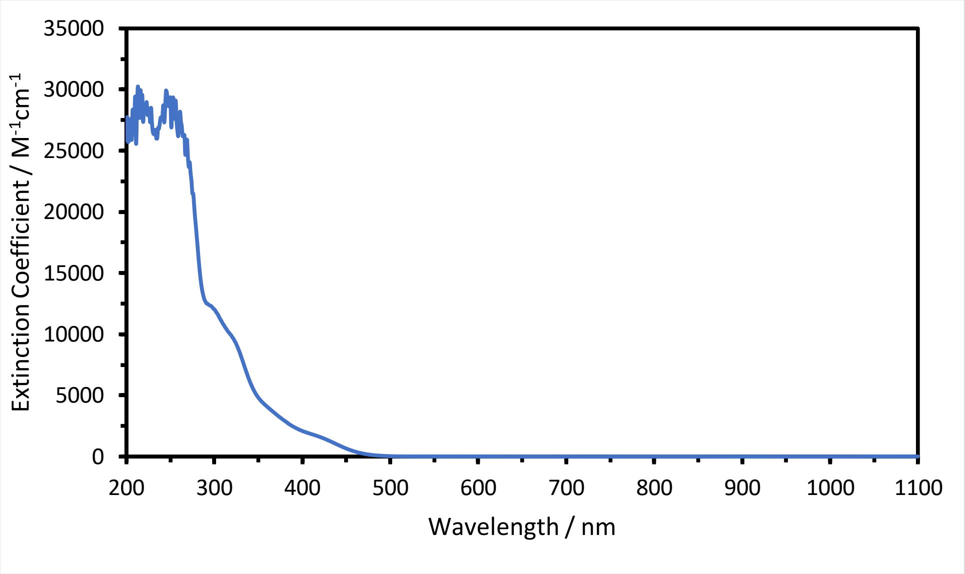

Figure S6: UV-Vis spectrum of [Cu(PY5)]OTf in anhydrous acetonitrile.

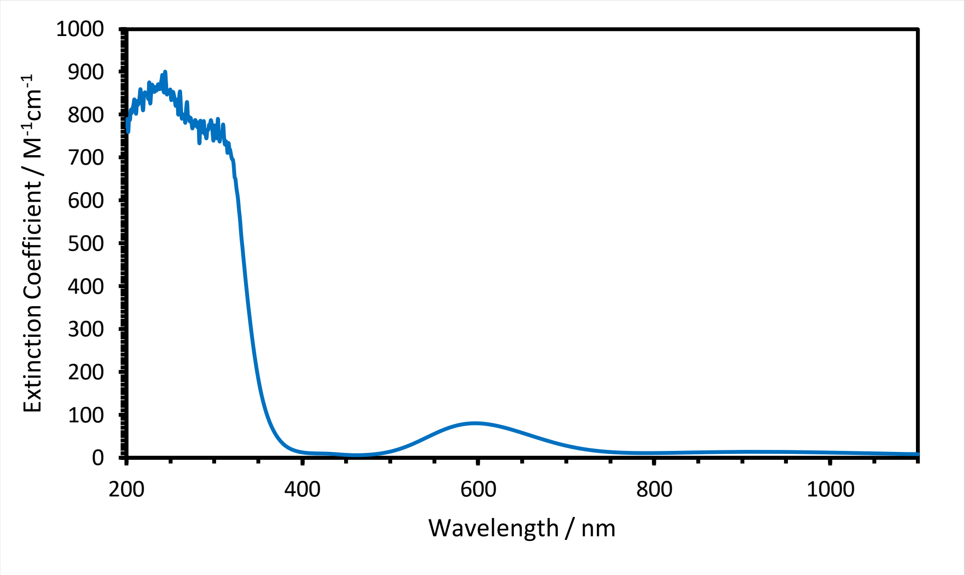

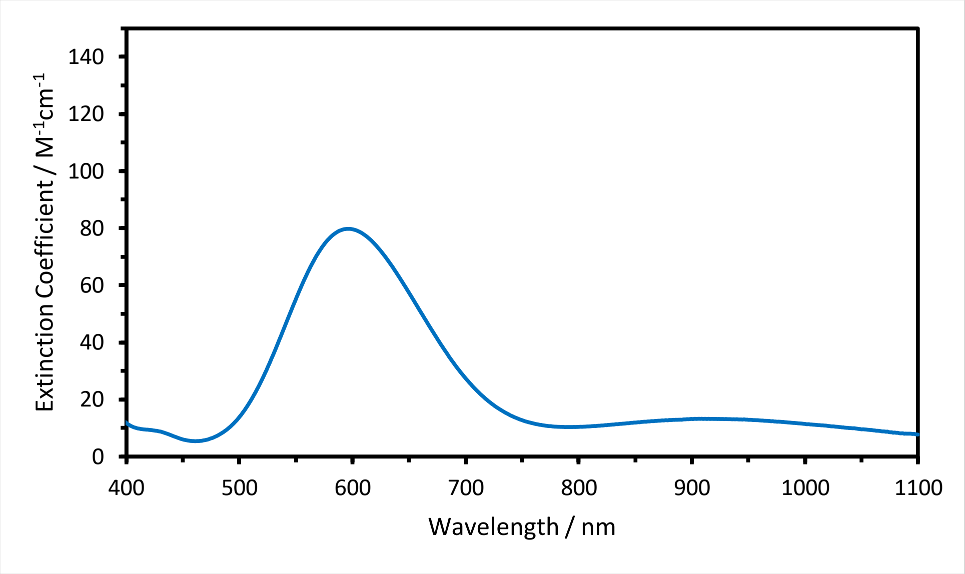

Figure S7: Top: UV-Vis spectrum of $[Cu(PY5)](OTf)_2$ in anhydrous acetonitrile. Bottom: expanded view to show d-d transitions.

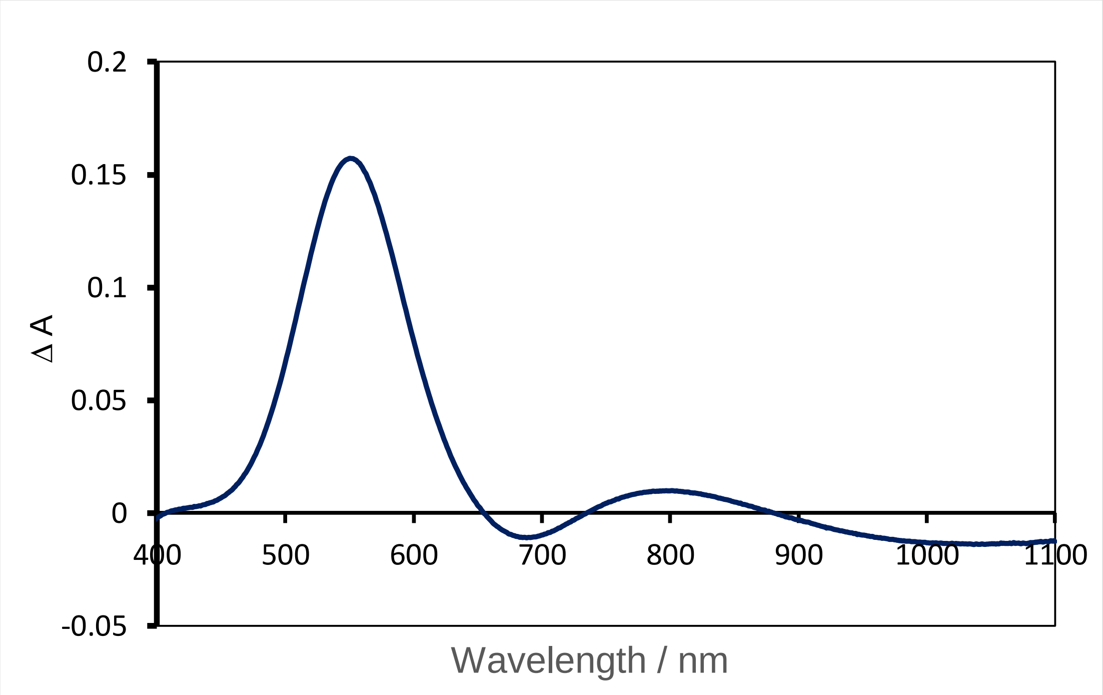

Figure S8: Difference UV-Vis spectrum: subtraction of spectra $[Cu(PY5)](OTf)_2$ from titrated solution with 10 equivalents of TBP in anhydrous acetonitrile, assigned as $[Cu(PY5)TBP](OTf)_2$

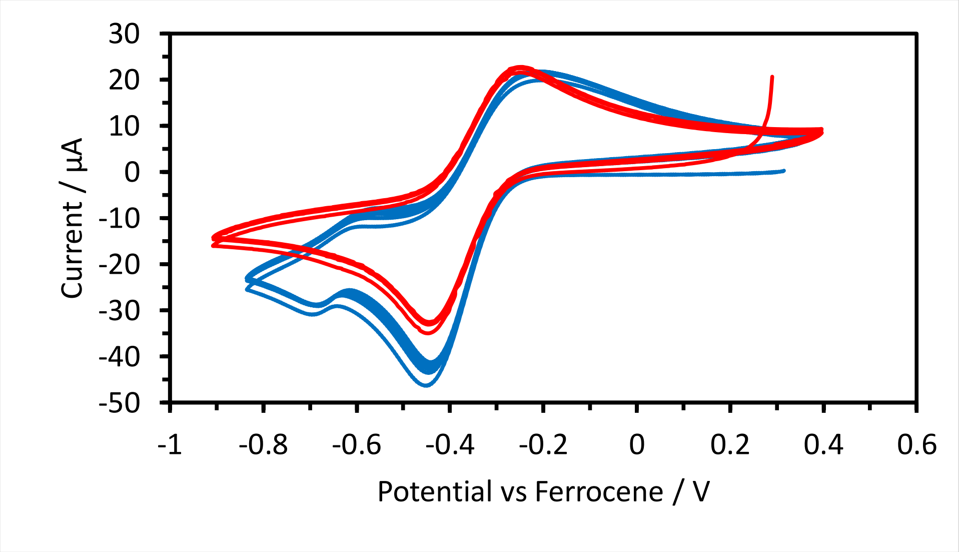

Figure S9: Cyclic Voltammogram of 2 mM of [Cu(PY5)]OTf$_2$, blue, and [Cu(PY5)]TFSI$_2$, red, in anhydrous acetonitrile containing 0.1 M LiX where X is the counter ion being investigated.

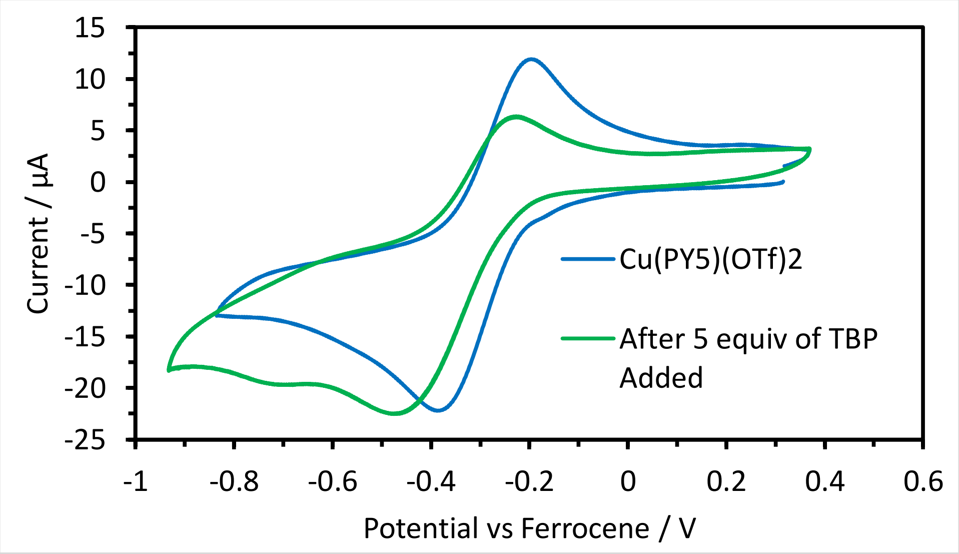

Figure S10: Cyclic Voltammogram of 2 mM of [Cu(PY5)]OTf$_2$ in dichloromethane, blue, with 5 equivalents of acetonitrile added to the solution, green. All samples contained 0.1 M TBAPF$_6$ as a supporting electrolyte, measured at a scan rate of 0.1 V/s on a glassy carbon working electrode.

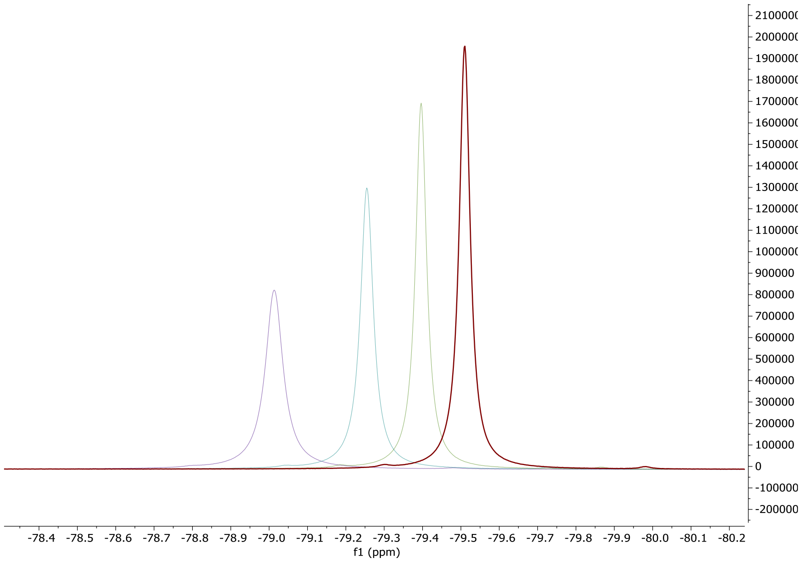

Figure S11: ^{19}F-NMR spectra of [Cu(PY5)]OTf$_2$ in anhydrous deuterated acetonitrile at various temperatures, 25°C-red, 0°C-green, -20°C-blue, and -40°C-purple. Full spectra were measured from -220 to 20 ppm but only one peak was found.

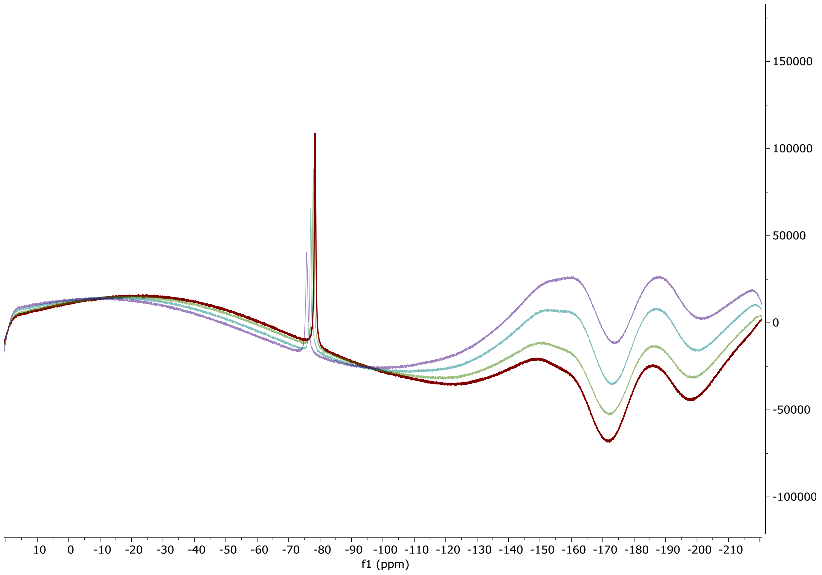

Figure S12: ^{19}F-NMR spectra of [Cu(PY5)]OTf$_2$ in deuterated dichloromethane at various temperatures, 25°C-red, 0°C-green, -20°C-blue, and -40°C-purple.

Table S1. Summary of device performance of devices containing [Cu(PY5)]OTf with various equivalents of TBP added, parameters represent the average of five devices.

	η / %	J_{sc} / mA cm^{-2}	V_{oc} / V	*FF*
0 M TBP	2.00(±0.06)	8.40(±0.09)	0.39(±0.00)	0.61(±0.02)
0.055 M TBP	2.12(±0.10)	8.91(±0.39)	0.41(±0.02)	0.58(±0.04)

Table S2: The measured current and the integrated current found via IPCE for devices containing [Cu(PY5)]OTf$_{1/2}$ and various equivalents of TBP added.

	J_{sc} / mA cm^{-2}	Integrated Current / mA cm^{-2}
0 M TBP	8.40(±0.09)	8.92
0.055 M TBP	8.91(±0.39)	9.82
0.526 M TBP	7.81(±0.63)	10.64

Stopped-Flow

Stopped-flow spectroscopy was utilized to measure the cross-exchange electron transfer rate constant, k_{12}, between [Cu(PY5)]OTf$_2$ and octamethylferrocene (FcMe8) following the work done by Xie et al.[1]

$$[Cu(PY5)]^{2+} + FcMe8 \xleftrightarrow{k_{12}} [Cu(PY5)]^{+} + FcMe8^{+}$$

Octamethylferrocene was chosen for the cross-exchange to optimize the driving force and because it has a well-known self-exchange rate constant for electron transfer.[2] Due to the large potential difference between $[Cu(PY5)]^{2+/+}$ and $[FcMe8]^{+/0}$, it was assumed that the reaction went to completion, with no significant back reaction. Figure S13 shows a fit of the absorbance at 450 nm vs time plot, which represents the growth of the $[Cu(PY5)]^{+}$ species in solution due to the reduction of $[Cu(PY5)]^{2+}$ by FcMe8, using the following equation:

$$A = A_{\infty} + (A_O - A_{\infty})e^{-k_{obs}t} \qquad (1)$$

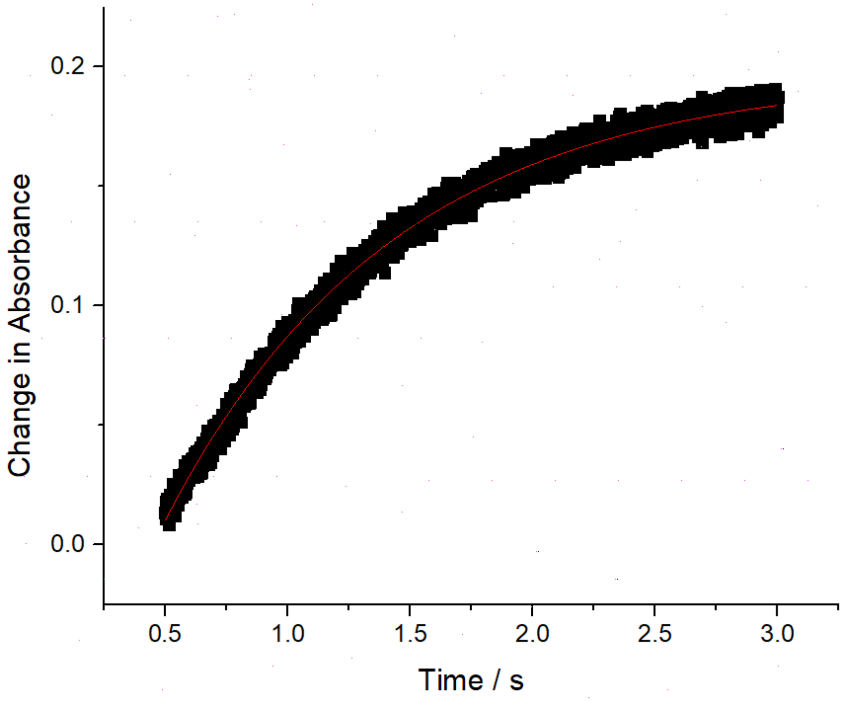

Figure S13: Plot of absorbance at 450 nm vs time, showing the increase of [Cu(PY5)]OTf$_2$ species (black dot) and fitting (red line) for the reduction of [Cu(PY5)]OTf$_2$ by octamethylferrocene (FcMe8).

For all reactions, FcMe8 was held in excess to maintain pseudo-first order conditions, allowing k_{obs} to be represented by:

$$k_{obs} = k_{12}[FcMe8] \tag{2}$$

A linear fit of the fitted k_{obs} values vs concentration of FcMe8 provided the value of the cross-exchange rate constant, k_{12}, from the slope of the line. The initial reaction mixtures and the pseudo-first order observed rate constants can be found in Table S2.

Table S3: The initial reaction mixture and the observed rate constant, k_{obs}, for the cross exchange between $[Cu(PY5)]OTf_2$ and FcMe8.

$[Cu(PY5)(OTf)_2]$ / M	[FcMe8] / M
	2.58×10^{-4}
	3.10×10^{-4}
2.74×10^{-5}	3.61×10^{-4}
	4.13×10^{-4}
	4.65×10^{-4}

Using the experimentally determined cross-exchange rate constant, k_{12}, and the previously determined self-exchange constant for $[FcMe8]^{+/0}$, k_{11}, the Marcus cross-relation

$$k_{12} = (k_{11}k_{22}K_{12}f_{12})^{1/2}W_{12} \tag{3}$$

was used to calculate the self-exchange rate constant for $[Cu(PY5)]^{2+/+}$, k_{22}. Where K_{12} is the equilibrium constant, f_{12} is a nonlinear correction term, and W_{12} is an electrostatic work term for bringing the reactants into contact. The nonlinear correction term and the work term were calculated (see below for details on the calculation) and determined to be 2.60 and 0.99, respectively (Table S4). However, it should be noted that there will be some error in the calculation of the work term since the Debye-Huckel model is not expected to have accurate results when the

solution has a high ionic strength, as was the case for this experiment (0.1 M supporting electrolyte). The equilibrium constant, K_{12}, can be determined using equation 5:

$$nF\Delta E = RT\ln K_{12} \tag{4}$$

where n is the number of electrons transferred, F is Faraday's constant, ΔE is the difference in formal potential between the oxidant and reductant in solution, R is the ideal gas constant, T is the temperature in Kelvin. The redox potential for $[Cu(PY5)]^{2+/+}$ was found to be -0.372 V vs ferrocene and $[FcMe8]^{+/0}$ has been previously determined to be -0.377 V vs ferrocene[3], respectively, using cyclic voltammetry which gave a calculated K_{12} of 0.62. The self-exchange rate constant for $[FcMe8]^{+/0}$ was previously identified to be 2.01×10^7 $M^{-1}s^{-1}$ using NMR techniques.[4] Using these values, the self-exchange rate for the $[Cu(PY5)]OTf_{1/2}$ couple was determined to be 88.1 (±7.3) $M^{-1}s^{-1}$.

Table S4: Summary of the kinetic data used to calculate the self-exchange rate constant of the $[Cu(PY5)]OTf_{1/2}$ complex by monitoring the reaction of $[Cu(PY5)]OTf_2$ and FcMe8 in acetonitrile containing 0.1 M LiOTf at 25°C.

Kinetic Parameter	Values
K_{12}	0.62
k_{12} / $M^{-1}s^{-1}$	4.53×10^4
k_{22} / $M^{-1}s^{-1}$	2.01×10^7
f_{12}	1
W_{12}	2.6
k_{11} / $M^{-1}s^{-1}$	88.1
λ_{se} / eV	2.57
λ_i / eV	1.55
λ_o / eV	1.02

The work required to move the reactant complexes to a distance, r, for the electron transfer reaction was calculated using the following equations:

$$W_{12} = \exp[-\frac{w_{12}+w_{21}-w_{11}-w_{22}}{2RT}] \quad (5)$$

$$w_{ij}(r) = \frac{z_i z_j q^2 N_A}{4\pi\varepsilon_0 \varepsilon r(1+\beta r)} \quad (6)$$

The above equation was used to determine the work associated with the forward and reverse cross-exchange reaction, w_{12} and w_{21} respectively, and the self-exchange reactions, w_{11} and w_{22}. In this equation z_i and z_j are the charges of the interacting complexes, q is the charge of an electron, N_A is Avogadro's constant, ε_0 is the permittivity of free space, ε is the static dielectric of the medium, $\beta = \left(\frac{2q^2 N_A I}{1000\varepsilon_0 \varepsilon k_B T}\right)^{1/2}$, I is the ionic strength of the solution and k_B is the Boltzmann's constant. The calculation has the following assumptions: the work is assumed to be primarily Coulombic, the distance, r, is assumed to be the center-to-center distance between the complexes and the reactants are assumed to be spherical.

The non-linear correction term, f_{12}, was calculated using the equation:

$$lnf_{12} = \frac{1}{4}\frac{\left(lnK_{12}+\frac{w_{12}-w_{21}}{RT}\right)^2}{\ln\left(\frac{k_{11}k_{22}}{Z^2}\right)+\frac{w_{11}+w_{22}}{RT}} \quad (7)$$

where K_{12} is the equilibrium constant, w_{12} and w_{21} are the work associated with the forward and reverse cross-exchange reaction respectively, R is the ideal gas constant, T is temperature in Kelvin, and Z is the frequency factor, which is assumed to be 10^{13} $M^{-1}s^{-1}$ due to the larger inner-sphere contributions to the total reorganization energy.

The total reorganization energy of the self-exchange reaction was calculated following the equation:

$$k_{11} = K_{12} Z \Gamma e^{-(\lambda_{11}/4k_B T)} \quad (8)$$

where k_{11} is the previously calculated self-exchange rate, K_{12} is the equilibrium constant, Z is the frequency factor, which is assumed to be 10^{13} $M^{-1}s^{-1}$ due to the larger inner-sphere contributions to the total reorganization energy, Γ is a correction for nuclear tunneling, which is assumed to be ~1, λ_{11} is the total reorganization energy of the self-exchange reaction, k_B is the Boltzmann constant, and T is the temperature of solution. The resulting λ_{11} was found to be 2.57 eV for the OTf complex and 2.41 eV for the TFSI complex.

The outer-sphere reorganization energy for two spherical reactants can be determined using

$$\lambda_{11,o} = \frac{(\Delta zq)^2}{4\pi\varepsilon_0}\left(\frac{1}{2a_1} + \frac{1}{2a_2} - \frac{1}{r_{11}}\right)\left(\frac{1}{D_{op,sol}} - \frac{1}{D_{s,sol}}\right) \tag{9}$$

where $\lambda_{11,o}$ is the outer-sphere reorganization energy, Δz is the change in charge of the complex, q is the charge of an electron, ε_0 is the permittivity of free space, a_1 and a_2 are the atomic radii of the reactants, r_{11} is the center–center distance between the reactants, $D_{op,sol}$ is the optical dielectric constant of the medium, which is equal to the square of the refractive index of the medium, and $D_{s,sol}$ is the static dielectric constant of the medium. The outer-sphere reorganization energy for the self-exchange reaction was calculated to be 1.02 eV for the OTf complex and 1.01 eV for the TFSI complex. The inner-sphere reorganization energy was determined using $\lambda_{11,i} = \lambda_{11} - \lambda_{11,o}$ yielding a result of 1.55 eV for the OTf complex and 1.40 eV for the TFSI complex for the inner-sphere reorganization energy of the self-exchange reaction. This inner-sphere reorganization energy is for a pair of $[Cu(PY5)]^{2+/+}$ molecules undergoing self-exchange electron-transfer reaction, $\lambda_{in,se}$, and thus λ_{in} ~ 0.74 eV for each donor / acceptor complex.

References

(1) Xie, Y.; Baillargeon, J.; Hamann, T. W. Kinetics of Regeneration and Recombination Reactions in Dye-Sensitized Solar Cells Employing Cobalt Redox Shuttles. *The Journal of Physical Chemistry C* **2015**, *119* (50), 28155–28166. https://doi.org/10.1021/acs.jpcc.5b08244.

(2) Raithel, A. L.; Kim, T.-Y.; Nielsen, K. C.; Staples, R. J.; Hamann, T. W. Low-Spin Cobalt(<scp>ii</Scp>) Redox Shuttle by Isocyanide Coordination. *Sustain Energy Fuels* **2020**, *4* (5), 2497–2507. https://doi.org/10.1039/D0SE00314J.

(3) Swarts, P. J.; Conradie, J. Redox Data of Ferrocenylcarboxylic Acids in Dichloromethane and Acetonitrile. *Data Brief* **2020**, *30*, 105650. https://doi.org/10.1016/j.dib.2020.105650.

(4) Baillargeon, J.; Xie, Y.; Hamann, T. W. Bifurcation of Regeneration and Recombination in Dye-Sensitized Solar Cells via Electronic Manipulation of Tandem Cobalt Redox Shuttles. *ACS Appl Mater Interfaces* **2017**, *9* (39), 33544–33548. https://doi.org/10.1021/acsami.7b01626.
